# External quality assessment demonstrates that PD‐L1 22C3 and SP263 assays are systematically different

**DOI:** 10.1002/cjp2.153

**Published:** 2019-12-17

**Authors:** Andrew Dodson, Suzanne Parry, Birgit Lissenberg‐Witte, Alex Haragan, David Allen, Anthony O'Grady, Emma McClean, Jamie Hughes, Keith Miller, Erik Thunnissen

**Affiliations:** ^1^ UK National External Quality Assessment Scheme for Immunocytochemistry and In‐Situ Hybridisation London UK; ^2^ Department of Epidemiology and Biostatistics Amsterdam UMC, Vrije Universiteit Amsterdam Amsterdam The Netherlands; ^3^ Department of Pathology Royal Liverpool and Broadgreen University Hospitals Liverpool UK; ^4^ HSL‐Advanced Diagnostics London UK; ^5^ Department of Pathology Royal College of Surgeons in Ireland, Beaumont Hospital Dublin Ireland; ^6^ Oncology, Haematology and Cellular Pathology Guy's and St Thomas' NHS Foundation Trust London UK; ^7^ Department of Pathology Amsterdam UMC, Vrije Universiteit Amsterdam Amsterdam The Netherlands

**Keywords:** external quality assessment, PD‐L1, predictive testing, immunohistochemistry, non‐small cell lung cancer, companion diagnostic assays

## Abstract

PD‐L1 inhibitors are part of first line treatment options for patients with advanced non‐small cell lung cancer. PD‐L1 immunohistochemistry (IHC) assays act as either a companion or a complementary diagnostic. The purpose of this study is to describe the experience of external quality assurance (EQA) provider UK NEQAS ICC and ISH with the comparison of different PD‐L1 assays used in daily practice. Three EQA rounds (pilot, run A and run B) were carried out using formalin fixed paraffin embedded samples with sample sets covering a range of epitope concentrations, including ‘critical samples’ near to clinical threshold cut‐offs. An expert panel (*n* = 4) evaluated all returned slides simultaneously and independently on a multi‐header microscope together with the participants own in‐house control material. The tonsil sample was evaluated as ‘acceptable’ or ‘unacceptable’, and for the other samples the percentage of PD‐L1 stained tumour cells were estimated in predetermined categories (<1%, 1 to <5%, 5 to <10%, 10 to <25%, 25 to <50%, 50 to <80%, 80 to 100%). In the pilot and the two subsequent runs the number of participating laboratories was 43, 69 and 76, respectively. The pass rate for the pilot run was 67%; this increased to 81% at run A and 82% at run B. For two ‘critical samples’, in runs A and B, 22C3 IHC had significantly higher PD‐L1 expression than SP263 IHC (*p* < 0.001), whilst the PD‐L1 scores for the other six samples were similar for all assays. In run A the laboratory developed tests (LDTs) using 22C3 scored lower than the commercial 22C3 tests (*p* = 0.01). After the initial testing, improvement in performance of PD‐L1 IHC is shown for approved and LDT PD‐L1 assays. Equivalency of approved PD‐L1 22C3 and SP263 assays cannot be assumed as the scores cross the clinically relevant thresholds of 1% and 50% PD‐L1 expression.

## Introduction

Non‐small cell lung carcinoma (NSCLC) is the most common cause of cancer‐related death in the world. NSCLC is histologically a heterogeneous group of cancers and, within the adenocarcinomas, different genetic changes are associated with different treatment modalities 1, 2. The recent introduction of immune checkpoint inhibitors has changed the standard of care for advanced and stage III NSCLC 3. For patients with advanced NSCLC without driver mutations, nivolumab, pembrolizumab and atezolizumab are available as second‐line treatments. For pembrolizumab the PD‐L1 assay is obligatory (companion diagnostic) while for the others the PD‐L1 test is optional (complementary diagnostic) 4. Pembrolizumab is also available for first‐line monotherapy, but only in patients with high (>50%) PD‐L1 expression. A further development is that, for NSCLC patients without *EGFR* or *ALK* mutations, PD‐L1/PD‐1 inhibition may be added to standard chemotherapy 5.

Most of the clinical trials involving these inhibitors have demonstrated an association between response rate, outcomes and amount of tumour cell PD‐L1 expression (tumour proportion score; TPS), determined by immunohistochemistry (IHC). Currently, five different IHC assays have been developed in conjunction with pharmaceutical companies 6.

Since the introduction of PD‐L1 as a predictive IHC biomarker, differences between diagnostic and clinical validation have become apparent 7. For *analytical/technical* validation of a diagnostic test the threshold of positivity is not relevant, whilst for *clinical* validation of a predictive test the threshold should be as close as possible to the test validated by clinical data. The latter is associated with a likelihood of response to a certain treatment. For optimal comparison, so called ‘critical samples’ with a PD‐L1 epitope concentration close to the threshold of this clinically validated test are useful 8.

In general, this can be achieved with external quality assessment (EQA) samples distributed by a provider to several centres to examine the performance of a test, that is, performed in daily pathology practice.

The purpose of this study is to describe the PD‐L1 experience of EQA provider UK NEQAS ICC and ISH when comparing different assays used in daily practice with sample sets covering a range of epitope concentrations, including critical samples.

## Methods

Three EQA rounds were carried‐out between March 2017 and January 2018 at approximately equally spaced intervals. There was an initial single pilot assessment that was used to formulate the assessment criteria, followed by a further two assessments, here designated as runs A and B.

Samples distributed for assessment consisted of formalin fixed paraffin embedded (FFPE) NSCLC tissue, reactive tonsil tissue and FFPE cell lines (Catalogue number: HD787. Horizon Discovery, Cambridge, UK 9). Samples consisted of NSCLC tumours with a range of expression levels for PD‐L1, and also a set of cell lines of known expression.

Participant laboratories were provided with two unstained slides (one as a spare) and requested to cut their in‐house control (not requested for the first pilot assessment) onto the same slides. The laboratory was then requested to perform their standard PD‐L1 IHC assay on these slides. Subsequently, the PD‐L1 stained slides were returned to UKNEQAS for assessment.

Expert panels of four assessors drawn from SP, AH, DA, AOG, EM and EK evaluated all returned slides (both UK NEQAS ICC and ISH samples together with the participants’ own in‐house control materials) simultaneously and independently on a multi‐header microscope. The tonsil sample was evaluated as either ‘acceptable’ or ‘unacceptable’, and each of the cell lines and tumour samples was visually assessed for the estimated percentage of PD‐L1 stained tumour cells present (TPS). These estimates were assigned to predetermined categories: (‘Bins’ of <1%, 1 to <5%, 5 to <10%, 10 to <25%, 25 to <50%, 50 to <80%, 80 to 100%). Finally, the assessment team provided a score for overall quality out of 5, where a score of ‘1’ indicated a completely uninformative preparation and a score of ‘5’ indicated the ideal staining result (see Table 1 for full categorisation). The mean of the four assessors formed the consensus score. In instances where there was a difference greater than 1 category between assessors, the assessment was reviewed by the panel, to harmonise to maximally 1 category difference.

**Table 1:** Consensus quality assessment score interpretation. Marks were lost for weak or false negative, false positive or inappropriate staining and morphological damage due to excessive pre‐treatment

Quality score	Quality category	PD‐L1 demonstration
5	Excellent	Staining of excellent quality, showing the expected level of expression
4	Acceptable	Staining of good quality, showing the expected level of expression (minor non‐significant improvements are possible)
3	Borderline Acceptable	Staining suitable for interpretation. Samples showing expected level of expression. However, some technical issues noted, significant improvements needed.
2	Unacceptable	Staining of unacceptable quality for clinical interpretation. Significant technical improvements needed.
1	Unacceptable	No or almost no specific staining seen. Significant technical improvements needed.

At the time of the assessment, four companion diagnostic (CDx) assays were available, based on the 22C3 and 28‐8 primary antibody clones (Agilent Dako, CA, USA) and the SP142 and SP263 primary antibody clones (Roche Tissue Diagnostics, Oro Valley, AZ, USA).

‘Gold standard’ slides stained by the commercial developers of the CDx assays were used as a reference to provide the PD‐L1 TPS (and percentage immune cells [ICs] for the SP142 clone). These values were used by the assessment team to set the scoring criteria including the overall quality score. Participants’ slides were assessed in accordance with the assay they had used. Slides stained with a lab‐devised protocol were assessed by comparing them to the validated assay of the same clone, where available; otherwise it was compared against 22C3.

Pathologists may favour the dark brown colour of the SP263 IHC assay over the lighter brown colour of the 22C3 detection system. In the UK NEQAS assessment, the laboratories with light brown staining and low intensity did not receive a low score, as long as any background staining was below the positive intensity level. At the other end of the spectrum, if negative control samples contained too much background staining, resulting in precipitation in the cytoplasm of tumour cells, this was evaluated as false positive.

Statistical analyses 10 were carried out by BL‐W using SPSS for Windows and Mac version 22 (IBM Corp., Armonk, NY, USA). The significance level was set at 0.05.

## Results

### PD‐L1 testing in three rounds

In the pilot and the two subsequent runs the number of participating laboratories was 43, 69 and 76, respectively. The performance of the participants deemed ‘Acceptable’, ‘Borderline’ or ‘Failure’ for the three runs is shown in Table 2A–C, respectively. The pass rate (‘Acceptable’ and ‘Borderline’) increased after the pilot run from 67% to 81% for run A and to 82% for run B. For runs A and B the distribution of the antibodies and test type (categorised as approved or LDT) is shown in Table 2D.

**Table 2:** (A) The performance in the initial pilot run for the different assays is shown with number of participants (percentage in brackets). (B) The performance in run A for the different assays is shown with number of participants (percentage in brackets). (C) The performance in run B for the different assays is shown with number of participants (percentage in brackets). (D) The distribution of the different antibodies and test types, categorised as ‘approved assay’ and ‘LDT’, is shown for runs A and B. The percentages in brackets indicate proportional usage for each run within that category of test

(A)				
Assay	Acceptable	Borderline	Fail	Total
Dako Agilent 22C3 assay	5 (72%)	1 (14%)	1 (14%)	7
Dako Agilent 22C3 LDT	1 (9%)	4 (36%)	6 (55%)	11
Dako Agilent 28‐8 assay	0 (0%)	1 (100%)	0 (0%)	1
Roche/Ventana SP263 assay	9 (70%)	2 (15%)	2 (15%)	13
Roche/Ventana SP142 assay	3 (100%)	0 (0%)	0 (0%)	3
SP142 LDT	0 (0%)	0 (0%)	2 (100%)	2
Other antibodies LDT	1 (17%)	2 (50%)	3 (33%)	6
Total	19 (44%)	10 (23%)	14 (33%)	43

(B)				
Assay	Acceptable	Borderline	Fail	Total
Dako Agilent 22C3 assay	11 (79%)	2 (14%)	1 (7%)	14
Dako Agilent 22C3 LDT	3 (6%)	5 (46%)	3 (18%)	11
Dako Agilent 28‐8 assay	2 (100%)	0 (0%)	0 (0%)	2
Roche/Ventana SP263 assay	21 (75%)	3 (11%)	4 (14%)	28
Roche/Ventana SP263 LDT	0 (0%)	0 (0%)	1 (100%)	1
Roche/Ventana SP142 assay	3 (100%)	0 (0%)	0 (0%)	3
Cell Signaling LDT	0 (0%)	3 (60%)	2 (40%)	5
Method unknown	1 (20%)	2 (40%)	2 (40%)	5
Total	41 (59%)	15 (22%)	13 (19%)	69

(C)				
Assay	Acceptable	Borderline	Fail	Total
Dako Agilent 22C3 assay	15 (88%)	0 (0%)	2 (12%)	17
Dako Agilent 22C3 LDT	6 (67%)	1 (11%)	2 (22%)	9
Dako Agilent 28‐8 assay	0 (0%)	2 (100%)	0 (0%)	2
Roche/Ventana SP263 assay	26 (84%)	3 (10%)	2 (6%)	31
Roche/Ventana SP142 assay	1 (25%)	2 (50%)	1 (25%)	4
Abcam PD‐L1 (28‐8) LDT	1 (33%)	0 (0%)	2 (67%)	3
Cell Signaling LDT	2 (67%)	1 (33%)	0 (0%)	3
Diagomics PDL1 QR1 LDT	0 (0%)	0 (0%)	1 (100%)	1
Method unknown	1 (17%)	1 (17%)	4 (67%)	6
Total	52 (68%)	10 (13%)	14 (18%)	76

(D)				
Run	Primary antibody	Approved assay	LDT	Total
A	22C3	14 (30%)	11 (65%)	25 (39%)
	28‐8	2 (4%)	0 (0%)	2 (3%)
	SP263	28 (60%)	1 (6%)	29 (45%)
	SP142	3(6%)	0 (0%)	3 (5%)
	E1L3N	0 (0%)	5 (30%)	5 (8%)
	Total	47	17	64
B	22C3	17 (32%)	9 (56%)	26 (37%)
	28‐8	2 (4%)	3 (19%)	5 (7%)
	SP263	31 (57%)	0 (0%)	31 (44%)
	SP142	4 (7%)	0 (0%)	4 (6%)
	E1L3N	0 (0%)	3 (19%)	3 (4%)
	QR1	0 (0%)	1 (6%)	1 (1%)
	Total	54	16	70

### Effect of epitope concentration

**Figure 1:**
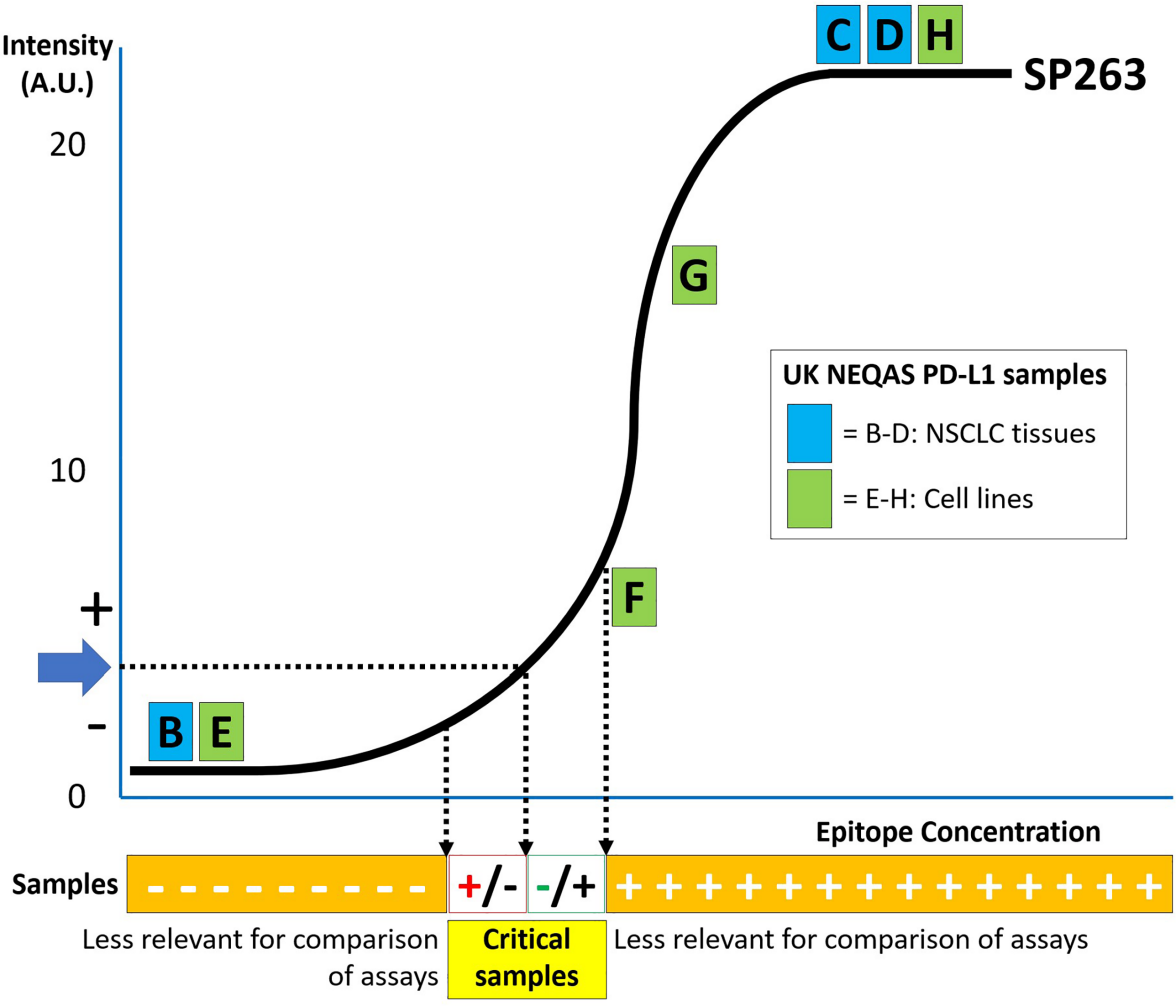
The relationship between epitope concentration and intensity is shown with the black line 35. Critical samples are positioned around a threshold of the IHC test 8. The approximate PD‐L1 intensities for 7 of the 8 distributed EQA samples (B–H) are shown along the black line (A, tonsil [not shown]). Note that samples outside the critical range are less likely to result in a different outcome, i.e., remain negative or positive.

**Figure 2:**
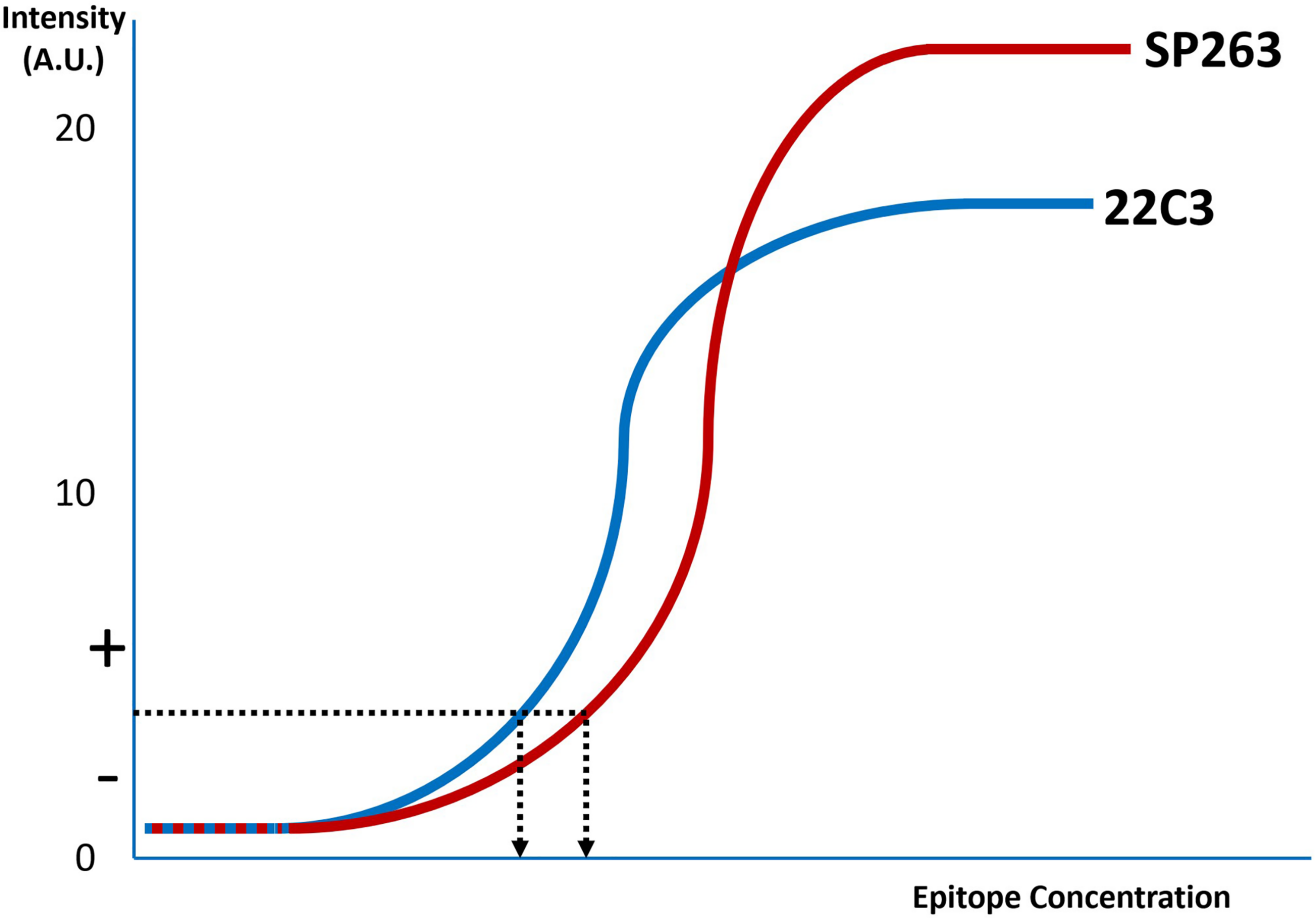
A graphical display of the lines representing the likely difference between SP263 and 22C3 approved tests. The arrows denote the threshold of the test distinction between positive (+) and negative (−). Note that the SP263 is positive at a slightly higher epitope concentration than 22C3.

The approximate PD‐L1 intensity of the distributed cell line and NSCLC tissue samples is shown in Figure 1. For the PD‐L1 unequivocally strong positive and negative cell lines (Figure 1, B–D) and NSCLC tissue samples (Figure 1, E and H) no significant difference in outcome was observed between the approved assays and LDTs for the different PD‐L1 clones, nor between the different approved assays themselves (data not shown, *P* values ranging from 0.1 to 1.0). For the weak positive PD‐L1 IHC sample (Figure 1, F), data for the two assays with highest frequency (22C3 and SP263) are shown in Table 3A and for the sample close to the strong positive plateau of IHC (Figure 1, G) in Table 3B. Note that for sample F as well as sample G, 22C3 had on average higher scores for PD‐L1 expression than SP263 (*p* < 0.001). In run A, for sample F, the LDT assays for 22C3 had lower TPS scores than the approved 22C3 assays. Figure 2 shows a graphical representation of the difference between the two assays.

**Table 3:** A. The distribution of PD‐L1 is shown for 22C3 and SP263 for the sample close to the threshold (F) in runs A and B (the figures in brackets indicate proportion of scores within that assay type). B. The distribution of PD‐L1 is shown for 22C3 and SP263 for the sample close to the strong positive plateau of IHC (G) in runs A and B (the figures in brackets indicate proportion of scores within that assay type). *P* values are shown for comparison between commercially approved assays (approved assay) and LDT and between the two approved assays using the 22C3 and SP263 antibodies respectively. n/a, not assessable

(A)									
Primary antibody	TPS	Approved assay	LDT	*P* value (approved assay/LDT)	Primary antibody	Approved assay	LDT	*P* value (approved assay/LDT)	*P* value (approved assay, 22C3/SP263)
Run A									
22C3	<1%	–		**0.01**	SP263	12 (43%)	–	0.069	<0.001
	1 to 4%	1 (7%)	7 (64%)			15 (54%)	–		
	5 to 9%	5 (36%)	1 (9%)			1 (4%)	–		
	10 to 24%	8 (57%)	3 (27%)			–	–		
	25 to 49%	–	–			–	–		
	50 to 79%	–	–			–	1 (100%)		
	80 to 100%	–	–			–	–		
Run B									
22C3	<1%	1 (6%)	–	0.85	SP263	51 (16%)	–	n/a	<0.001
	1 to 4%	1 (6%)	2 (22%)			20 (65%)	–		
	5 to 9%	3 (18%)	1 (11%)			2 (6%)	–		
	10 to 24%	11 (65%)	6 (67%)			3 (10%)	‐		
	25 to 49%	1 (6%)	–			1 (3%)	–		
	50 to 79%	–	–			–	–		
	80 to 100%	–	–			–	–		

(B)									
Primary antibody	TPS	Approved assay	LDT	*P* value (approved assay/LDT)	Primary antibody	Approved assay	LDT	*P* value (approved assay/LDT)	*P* value (approved assay, 22C3/SP263)
Run A									
22C3	<1%	–	–	0.15	SP263	–	–	0.034	<0.001
	1 to 4%	–	–			–	–		
	5 to 9%	–	–			–	–		
	10 to 24%	–	–			4 (14%)	–		
	25 to 49%	–	3 (27%)			12 (43%)	–		
	50 to 79%	10 (71%)	7 (64%)			12 (43%)	–		
	80 to 100%	4 (29%)	1 (9%)			–	1 (100%)		
Run B									
22C3	<1%	–	–	0.74	SP263	–	–	n/a	<0.001
	1 to 4%	–	–			–	–		
	5 to 9%	–	–			–	–		
	10 to 24%	1 (6%)	–			–	–		
	25 to 49%	0 (0%)	–			5 (16%)	–		
	50 to 79%	2 (12%)	2 (22%)			23 (74%)	–		
	80 to 100%	14 (82%)	7 (78%)			3 (10%)	–		

## Discussion

This study reports an improvement in performance of PD‐L1 IHC after the initial testing for approved as well as LDT assays. For the samples with an epitope concentration close to the threshold of the clinically validated test, the SP263 stained repeatedly fewer tumour cells than the comparator IHC assay 22C3. Importantly, these differences cross the clinically relevant thresholds of 1% and 50% PD‐L1 expression.

Our study clearly shows the effect of epitope concentration in terms of informative and not‐informative (negative or strongly PD‐L1 positive) samples for comparison of PD‐L1 IHC assays. In the studies examining the concordance rate of different PD‐L1 assays the composition of the case set is crucial. The College of American Pathologists (CAP) 11 and Clinical & Laboratory Standards Institute 12 guidelines define that laboratories should achieve at least 90% overall concordance between a new test and the comparator test. If concordance is less than 90%, laboratories need to investigate the cause of low concordance. The *a priori* chance for 90% concordance will be high if 55% PD‐L1 negative samples 13 and 35% PD‐L1 strongly positive samples are examined 14. The composition of the case set varies from resection specimens only, to a mixture of small samples (with or without cytology) and resection specimens. In all these studies the samples were based on preferential selection. For indirect clinical validation one may argue that the 90% concordance between two IHC tests should hold in a series of consecutive cases, which is not a requirement for diagnostic validation.

This study demonstrates that PD‐L1 expression in two samples close to the threshold repeatedly stains differently between approved SP263 and 22C3 assays. This seems in contrast to several studies comparing PD‐L1 assays, where the message is put forward that 28.8, 22C3 and SP263 perform similarly 15, 16, 17. However, a note of caution holds for the Blueprint 2 study: the supplementary data for that study show only a concordance rate of 84% (43/51) between SP263 and 22C314. Taking the CAP guidelines into account this does not support the conclusion from the authors of ‘analytical evidence for interchangeability of the 22C3, 28‐8 and SP263 assays’. Remarkably, other studies reported that SP263 had higher PD‐L1 scores than 22C3 in several cases 18, 19 or low correlation between these two assays 20, while Kim and colleagues show more positive cases in 22C3 than in SP263 21. Interestingly, when looking at individual cases around the threshold of 1% PD‐L1 positivity, comparison of 22C3 with SP263 shows in some cases higher staining for SP263 and in other cases higher for 22C3 16, 17. Although the PD‐L1 assays target the same protein, the test performance is different at least in some cases.

The statement ‘SP263 and 22C3 are interchangeable’ is not demonstrably refuted by this study but remains in question at least until a series of consecutive cases large enough in power to provide significant findings have been examined. It is of note that, based on the test used, patients are likely to be treated differently at least on the basis of some samples.

Our report also shows a learning effect over time for PD‐L1, which is in line with the reports of NordiQC 22, 23. False positivity is, in UK NEQAS ICC and ISH and NordiQC EQA experience, rare and false negativity is more frequent in approved assays as well as LDTs 23, 24.

Factors affecting comparability between PD‐L1 assays include the following: (1) different antibody clones will target different epitopes 25, information about which may be proprietary and mostly not publicly available; (2) different detection systems 26 and automated staining platforms will affect comparability 27; (3) post translational modification may be of influence 28; (4) preanalytical factors, such as variation in type of fixative and duration of fixation can significantly influence the outcome of IHC procedures 29. Delay in fixation has been repeatedly shown to affect the retention of certain proteins within tissues. One early report from the Dowsett group working with phospho‐proteins in breast cancer tissues showed that there was an almost complete loss of reactivity for p‐AKT and p‐ERK in breast resection tissues in contrast to strong staining seen in matched core biopsies from the same samples 30. Moreover, in a NSCLC model, delayed formalin fixation (increases in time before the start of fixation) caused significant loss of immunoreactivity 31: for PD‐L1, there was a reduction in the proportion of tumour cells showing positive membrane staining and the effect was larger with increase in fixation delay. In addition, (5) methods used to decalcify bony tissues and thus enable the preparation of sections from them can also significantly affect IHC results. This is particularly relevant in the context of PD‐L1 demonstration in NSCLC as bone is a frequent site for metastasis. Both acid and ethylenediaminetetraacetic acid (EDTA) declacifiers reduce the proportion of PD‐L1 positive cells stained and the intensity with which they stain. Acid decalcification is more deleterious and shows an effect sooner than EDTA 32.

The tissues and cell lines selected by UK NEQAS ICC and ISH for use in their EQA programmes were rigorously assessed and validated prior to their use. In regard to the PD‐L1 NSCLC module, testing was conducted using two anti‐PD‐L1 clones (SP263, Ventana and 22C3, Agilent Dako) in a number of laboratories. Sections from multiple levels through the EQA blocks were stained to examine and control for heterogeneous expression.

For multiple examined antigens across a variety of modules, time delay between sectioning by UK NEQAS and staining in the participant laboratories has been examined for its effect on staining results with the consistent finding that, provided sections are stained by the participant soon after receipt, deterioration is not present or is negligible (unpublished observations AD, SP) and is in line with literature on stability of histological slide preparations 33.

Tissues with high epitope concentration may be positive in all tests. The most prominent example is placenta, an intended positive control in at least one US Food and Drug Administration (FDA) approved assay 34. In the UK NEQAS PD‐L1 EQA schemes, so far, negative PD‐L1 IHC staining of placenta has not been seen, even in the instances where cases have failed due to a relatively low PD‐L1 epitope concentration.

In summary, after initial testing, improvement in performance of PD‐L1 IHC is documented for approved PD‐L1 assays as well as LDTs. As most diagnostic laboratories prefer to run only one PD‐L1 IHC assay on often very limited tissue samples, the use of interchangeable assays would be advantageous. However, this EQA study shows that equivalency of approved PD‐L1 assays cannot be assumed.

## Author contributions statement

AD, SP and ET wrote the manuscript and AH critically revised it. SP collated and analysed the data. BL‐W provided data analysis. DA, TOG, EM and JH contributed to the assessment of assay results.
